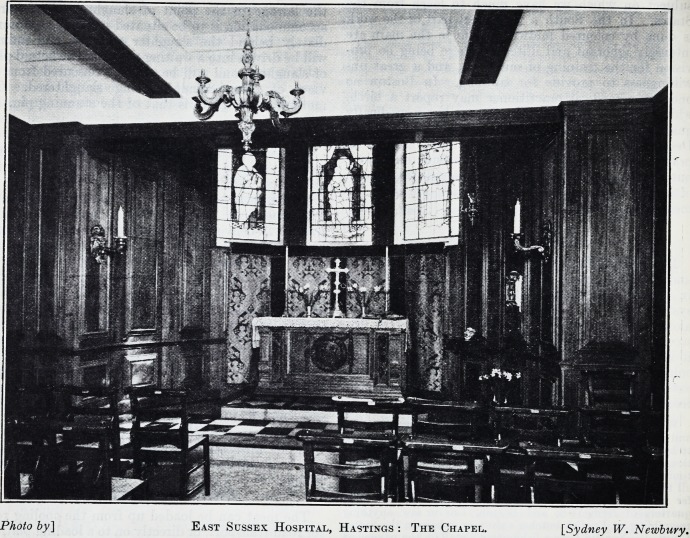# A Hospital Chapel

**Published:** 1924-10

**Authors:** 


### A HOSPITAL CHAPEL.

The East Sussex Hospital at Hastings has recently moved from the Parade to a delightful new building, which stands above the White Rock Gardens, with a fine view overlooking the sea. It now possesses a beautiful little chapel for the patients and nurses, a photograph of which we reproduce. The hospital much needed a chapel in the actual building for both the staff and the patients, as it is a considerable distance to a neighbouring church. After a great deal of consideration, Dr. Christopherson, the senior surgeon, was not satisfied until he took a leading hand in arranging for a chapel inside the precincts. Money was collected, the bulk of it being found by the Garnier family in memory of a son who was killed in the war, and numerous gifts, such as the altar cross, candlesticks and vases, altar frontal and stained glass windows, were given by individuals as memorials.

The room in which the chapel has been arranged was originally intended for a dispensary, but it lent itself wonderfully well for a chapel with an apsidal end, and a little room leading out of it which makes an admirable little vestry for the priest. The whole of the walls are clothed with rich panelling in walnut in the Georgian style, and at the east end three windows are filled with stained glass, given in memory of a late nurse of the hospital. Over the altar is a dossal of blue and gold tapestry, and the altar itself is richly carved, having in the centre the *Agnus Dei.* The chapel is lighted from an Italian gilded chandelier, and hidden lights give a pleasant subdued light. The footpace and communicants' step are arranged in ebony and oak squares, inlaid. The whole of the woodwork was carried out by Mr. Robinson, of 4 Bennett's Yard, Marsham Street, S.W., from the designs of Mr. Cecil G. Hare, of 11 Gray's Inn Square, W.C. 1.

*Photo by*] EAST SUSSEX HOSPITAL, HASTINGS: THE CHAPEL. [*Sydney W. Newbury.*

### HEALTH OF AMERICAN FACTORY WORKERS.

Investigation by the Edison Electric Illuminating Company of Boston regarding sickness and accidents in their works shows that sickness caused twenty times as many cases of absenteeism as accidents, and was responsible for seven times as much loss of time from work. The medical staff were chiefly occupied in the treatment or prevention of colds and bronchial troubles, digestive disturbances and "nerves." Men, it was found, suffered more from indigestion than women—an experience of no little interest in view of the fact that dyspepsia is often regarded as a woman's rather than a man's ailment. The firm has set itself to attempt to teach its workers some elementary truths about healthful eating.

**"To Wives and Mothers."**

**A sixth edition, revised, has been issued by the National League of Health, of their excellent pamphlet entitled, "To Wives and Mothers: How to Keep Yourselves and Your Children Well and Strong." It is published at the very moderate cost of fivepence.**

## Figures and Tables

**Figure f1:**